# Nightmare distress mediated the correlation between autobiographical memory specificity and depression

**DOI:** 10.1371/journal.pone.0318661

**Published:** 2025-02-25

**Authors:** Jiaxi Wang, Haote Fu, Xiaoling Feng, Heyong Shen

**Affiliations:** 1 Research Center for Embodied Cognition, School of Education, Guangzhou University, Guangzhou, China; 2 School of Psychology, South China Normal University, Guangzhou, China; 3 Institute of Analytical Psychology, City University of Macau, Macau, China; University of Pittsburgh School of Medicine, UNITED STATES OF AMERICA

## Abstract

In this study, we explored whether nightmare distress mediated the correlation between autobiographical memory specificity and depression. 112 participants provided their most recent dreams that happened within one month, and finished some scales that measured depression, autobiographical memory specificity, and nightmare distress. In line with our hypothesis, nightmare distress was the mediator that played a role in the relationship between autobiographical memory specificity and depression. In addition, we found that both nightmare distress and autobiographical memory specificity were correlated with the impact of a dream on one’s life story. By contrast, contrary to our hypothesis, we did not find any correlation between autobiographical memory specificity, or nightmare distress, and dream bizarreness. Potential implications from these results were discussed.

## 1. Introduction

### 1.1. Autobiographical memory specificity and depression

Autobiographical memory refers to an individual’s memory of personal life experiences. It contains autobiographical knowledge (such as personal conceptual knowledge) and episodic memories [1]. One characteristic of autobiographical memory is the specificity. Typically, specific autobiographical memories are memories related to a specific place and time, and their duration is not more than a day.

Clinical research suggests that compared with healthy individuals, depressed individuals tend to display non-specific autobiographical memories, which are more vague, summarizing experiences rather than concrete, detail-rich experiences [2]. For instance, when asked about times they felt happy, a depressed individual might respond with “I have never felt joy” rather than recalling a specific occasion of happiness. The correlation between depression and autobiographical memory specificity has been explained by different kinds of factors [2]. For example, avoiding specific autobiographical memories may reduce emotional distress caused by negative memories. This kind of explanation focuses on the effect of depression on autobiographical memory specificity. Yet some clinial evidence suggests that the latter variable can also affect the former variable, because interventions targeting impairments in reduced autobiographical memory specificity can improve depression [3,4]. To our knowledge, it was unclear that why the autobiographical memory specificity could affect depression. In this study, we aimed to explore this topic.

### 1.2. Nightmare distress and depression

Nightmares are disturbed dreams, often accompanied by intensely negative emotions and anxiety. The nightmare may not only wake up an individual, but also be continue to negatively affect the individual’s waking life. For example, nightmare severity was related to worry which refers to negatively thinking about future situations of a person’s life [5]. Many studies have found a close relationship between nightmares and a range of psychological disorders, including depression, anxiety disorders, and post-traumatic stress disorder [6]. Furthermore, some evidence suggests that nightmares may affect depression. For example, treating nightmares led to moderate reductions in depression [7]. This kind of evidence implies that nightmare distress plays a role in the formation of depression.

### 1.3. Nightmare distress and autobiographical memory specificity

In our view, autobiographical memory specificity may affect nightmare distress. This effect may be explained by two kinds of hypotheses (see below). One was related to the impact of dreams on one’s life story, and the other was related to dream bizarreness.

#### 1.3.1. The impact of dreams on one’s life story, autobiographical memory specificity, and nightmare distress.

Life story refers to a narrative-like account of a person’s life, and culturally generated knowledge of a person’s life, such as an ideal work [8]. It stands at the most abstract level of the autobiographical knowledge which is stored at a hierarchical structure [9]. The life story is related to personal identity, which is evolved from narratives that people constructs about one’s life [10]. Specific autobiographical memories may provide past cognitive-affective information that can be used to deal with current situations [11], and thus autobiographical memory specificity may be related to the adaptive functioning, which in turn affects the narratives of the life story. Similarly, as dreams contain narratives that are composed of autobiographical memories [12], dreams may also affect the life story.

In our view, individual differences in autobiographical memory specificity may affect the impact of dreams on one’s life story, because sometimes the effect of specific autobiographical memories on the life story may decrease the effect of dreams on the life story. Besides, nightmares are disturbing dreams that may cause negative effects on the life story. As there may be individual differences in the aspect of the impact of dreams on one’s life story, nightmare distress may be correlated with the impact of dreams on one’s life story.

As individual differences in autobiographical memory specificity may affect the impact of dreams on one’s life story, which in turn may be related to nightmare distress, the former variable may affect nightmare distress.

#### 1.3.2. Dream bizarreness, autobiographical memory specificity, and nightmare distress.

Dream bizarreness refers to dream content that manifests anomalous or incoherent combinations of perceptual features, or appears in inappropriate contexts or rarely in everyday life. Some dreams are more bizarre than others, as these bizarre dreams may be more unusual, or irrational [13]. The dream bizarreness may be related to the offline spreading activation process of autobiographical memories [12]. Specifically, the process contains two steps. Firstly, elements of different autobiographical memories are activated during sleep. Secondly, these elements may be put together to create a narrative in a dream. Dream bizarreness may reflect the joining together of disparate elements of different memories.

In our view, individual differences in autobiographical memory specificity may affect dream bizarreness. The reason was in the following: Takano et al. (2017) [14] found that reduced autobiographical memory specificity enhanced the activation of unassociated distractors during the spreading activation process of autobiographical memory network. This effect may be continuous to dreaming, as the continuity hypothesis of dreaming suggests that there is continuity, such as cognitions, emotions, and waking-life experiences, between waking life and dreaming [15–19]. As dream bizarreness may be related to the offline spreading activation process of autobiographical memory network (see above), individual differences in autobiographical memory specificity may affect dream bizarreness.

In addition, previous research may imply that nightmare distress is related to bizarre dreams [20]. Specifically, Robert & Zadra (2014) [20] compared the bizarreness and the emotionality among nightmares, bad dreams, and everyday dreams. The nightmares and the bad dreams were defined as disturbing dreams, while the everyday dreams were not. The difference between the nightmares and the bad dreams was that the former dreams cause individuals to wake up, while the latter dreams do not. Results showed that the nightmares were more bizarre and emotional than the bad dreams, which in turn were more bizarre and emotional than the everyday dreams. As the emotionality of both the nightmares and the bad dreams may predict the distress of the two kinds of dreams, the nightmares may cause more distress than the bad dreams. As the former dreams were more emotional and bizarre than the latter dreams, nightmare distress may be related to dream bizarreness.

As individual differences in autobiographical memory specificity may affect dream bizarreness, which in turn may be related to nightmare distress, the former variable may affect nightmare distress.

### 1.4. Aims

In this study, we aimed to explore potential mechanism which lived behind the effect of autobiographical memory specificity on depression (see section 1.1). As autobiographical memory specificity may affect nightmare distress (see below), and nightmare distress may lead to depression (see section 1.2), in our opinion, nightmare distress may be a factor that mediates the effect of autobiographical memory specificity on depression. Here we explored this topic.

In addition, as there was a lack of explanation for the potential effect of autobiographical memory specificity on nightmare distress, we explored whether the impact of dreams on one’s life story or dream bizarreness would be related to autobiographical memory specificity, or nightmare distress (see section 1.3). We explored the topics, because they may help to explain the potential mediating effect mentioned above.

## 2. Methods

### 2.1. Participants

154 participants completed the full online questionnaire to finish the measurement of depression, nightmare distress, and report their autobiographical memories, as well as their most recent dreams. Of them, 11 participants provided invalid answers which meant scores for all items were same or reported a higher nightmare frequency than dream frequency. 5 participants failed to record their autobiographical memories, or dreams, or both. 26 participants reported dreams that happened more than one month.

As a result, the data of 112 participants (48 females) were used for further analysis, as they provided valid data and their dreams happened within one month. The average age of participants were 22.62 (SD =  3.95), with an age range of 18–40. The local research ethics committee approved this study. The recruitment period for the study was from 2024.7.1 to 2024.7.15, and all subjects gave written informed consent before the start of the study.

### 2.2. Material

#### 2.2.1. The Beck Depression Inventory-II (BDI-II).

The BDI-II is a 21-item, 4-point scale [21]. It is used to measure the degree of depression within past two weeks. The score of each item has a range from 0 to 3, and thus the total score of the BDI-II ranges from 0 to 63. Higher score indicates worse depression. Here we used the Chinese version of the BDI-II [22].

#### 2.2.2. The Nightmare Disorder Index (NDI).

The NDI is a 5-item, 5-point self-report measure of DSM-5 nightmare disorder [23]. The score of each item has a range from 0 to 4, and thus the total score of the NDI ranges from 0 to 20. Higher score indicates more serious nightmare distress. Here we used the Chinese version of the NDI [24].

#### 2.2.3. The Autobiographical Memory Test (AMT).

The AMT is a cue-word paradigm, which instructs participants to recall autobiographical memories that are related to the cue-word [25]. Specifically, each time participants are given a cue word, they were instructed to retrieve a specific personal memory in response to the cue word within 1 minute. Williams & Broadbent (1986) [25] adopted ten emotional cue words. Half of them were pleasant words (e.g., happy, safe, etc.), while the others were unpleasant words (e.g., sorry, angry, etc.).

In this study, as participants were instructed to report not only autobiographical memories, but also dreams, we used six cue words in the AMT, which contained three pleasant words (pleasant, happy, joyful), and three negative words (panic, anxiety, nervous). The instruction for the AMT was the following: There are six cue words that may be related to some past memories of you. Please recall one past memory you can remember each time you see a cue word, and write down the memory as fully as you can.

#### 2.2.4. The most recent dream.

The method to collect dream was to ask participants to report a most recent dream, which was similar to Wang & Feng (2024) [26]. “Please write down the last dream you remember having, i.e., your most recent dream. This could be as recent as last night or from as far back as childhood but should be the most recent one you can remember having, no matter how long or short it is. Please describe this dream exactly and as fully as you remember. Your report should contain, whenever possible: a description of the setting of the dream, whether it was familiar to you or not; a description of the people, their age, sex, and relationship to you; any animals that appeared in the dream. If possible, describe your feelings during the dream and whether it was pleasant or unpleasant. Be sure to tell exactly what happened to you and the other characters in the dream”.

In addition, participants were instructed to rate the emotionality of the dream, the importance of the dream, and the impact of the dream on one’s life, by a 5-point-Likert-scale.

### 2.3. Procedure

Participants finished an online questionnaire, where they completed the BDI-II, NDI, AMT, and reported their most recent dreams (see section 2.2). In addition, participants were instructed to rate the emotionality of the dream, the importance of the dream, and the impact of the dream on their life, by a 5-point-likert-scale. As a reward, they could get a few money. Finally, two blind external judges rated the autobiographical memory specificity measured by the AMT (see section 2.4.1), and the dream everydayness and the dream rationality (see section 2.4.2). The cronbach’s consistency coefficients (α) for the rating process were from 0.72 (dream everydayness) to 0.86 (autobiographical memory specificity). All inconsistent ratings were discussed later until reaching an agreement. Statistical analyses were performed by the SPSS software.

### 2.4. Coding standard for external judges

#### 2.4.1. Autobiographical memory specificity.

The rating standard for external judges to rate autobiographical memory specificity was similar to WenZel et al. (2004) [27]: “*a memory was coded as specific if it referred to a discrete event that occurred during a period of time of no more than 1 day (cf. Williams & Broadbent, 1986). A memory was coded as general if it contained one or more of the following attributes: (a) there was not enough information provided to discern whether the person was referring to a discrete event that occurred in a 1 day period (e.g., “studying for an exam”); (b) an event that took place over the course of more than 1 day (e.g., “summer vacation”); or (c) a recurrent event (e.g., “every time I have to give a speech”).*”

A score of 0 to represent the general memory and a score of 1 to represent specific memory were used.

#### 2.4.2. Dream everydayness and dream rationality.

Similar to Robert & Zadra (2014) [20], the bizarreness of dreams was measured by the following two scales: “*The Rationality Scale measures the likelihood of occurrence of the dream content and the degree of his adherence to natural laws whereas the Everydayness Scale examines the degree to which the dream content approximates that of everyday life*”. Detailed rating scales are shown in appendix. The scales came from Cann & Donderi (1986) [13]. Yet rating scores of the two scales in Robert & Zadra (2014) [20] were used as continuous variables, while the scores in Cann & Donderi (1986) [13] were further processed into categorical variables. Here we adopted a similar score standard as Cann & Donderi (1986) [13]. A score of 0 to represent an everyday dream or a rational dream, and a score of 1 to represent a non-everyday dream or a non-rational dream were used.

### 2.5. Data analysis

The depression was measured by the total score of the BDI-II. The nightmare distress was measured by the total score of the NDI. The autobiographical memory specificity was measured by the total score of the AMT, which measured by summing the judges’ rating scores. In addition, the bizarreness of dreams was measured by a categorial way, in which a non-everyday dream or a non-rational dream would represent a bizarre dream, otherwise it would be a non-bizarre dream. In addition, we calculated the impact of dreams on one’s life story, by summing the emotionality of the most recent dream, the importance of the most recent dream, and the impact of the most recent dream on one’s life. Because these variables can measure the impact of dreams on one’s life story, since Fitzgerald and Broadbridge (2013) [28] suggests that emotionality, significance, and consequences are items that measure a latent structure, which is called “impact”, in autobiographical memory studies.

For the mediating effect of nightmare distress on the relationship between autobiographical memory specificity and depression, firstly, we explored the effect of autobiographical memory specificity on depression, and then we explored the effect of autobiographical memory specificity on nightmare distress, and then we explored the effect of both autobiographical memory specificity and nightmare distress on depression. The regression analysis was used.

In addition, based on the average score of autobiographical memory specificity, we divided participants into a low autobiographical memory specificity group, and a high autobiographical memory specificity group, and then we explored whether the former group’s dreams had more impacts on one’s life story than the latter group’s dreams, and whether the former group had more bizarre dreams than the latter group. Also, we explored whether there was a correlation between dream bizarreness or the impact of dreams on one’s life story and nightmare distress. The Chi-square analysis, the Wilcoxon rank-sum test and the Spearman correlation were used.

## 3. Results

Cronbach’s α of the whole BDI-II was 0.96, and Cronbach’s α of the whole NDI was 0.85. In addition, the Cronbach’s α of the emotionality of the most recent dream, the importance of the most recent dream, and the impact of the most recent dream on one’s life was 0.70. The descrptive data of depression, nightmare distress, autobiographical memory specificity, dream length, and the impact of dreams on one’s life story were in Table 1. The frequency of bizarre dream was 51.8%.

**Table 1:** The descriptive data of variables.

Category	Average (SD)
Dream length^a^	74.92 (62.19)
depression^b^	44 (15.01)
Nightmare distress^c^	7.02 (3.85)
Autobiographical memory specificity^d^	1.78 (1.73)
The impact of dreams on one’s life story^e^	9.82 (2.51)

^a^Only the number of words were counted

^b^4-point scale (0 to 3), range from 0 to 63

^c^5-point scale (0 to 4), range from 0 to 20

^d^Rated by judges, range from 0 to 6

^e^5-point scale (1 to 5), range from 3 to 15

**Fig 1:**
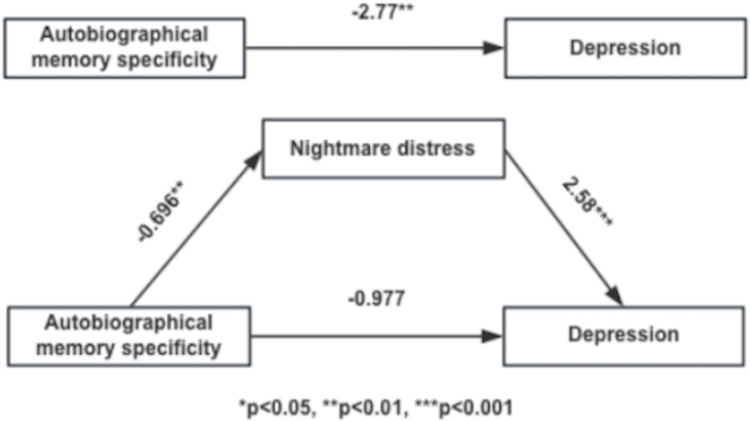
The mediating model of the mediating effect.

Regression analyses showed that autobiographical memory specificity significantly predicted depression, *β* =  -2.77, t =  3.53, *p* =  0.001, *R*^*2*^ (adjust) =  0.093; Similarly, autobiographical memory specificity significantly predicted nightmare distress, *β* =  -0.696, t =  -3.45, *p* =  0.001, *R*^*2*^ (adjust) =  0.089. In addition, when both autobiographical memory specificity and nightmare distress were used as indepedent variables, nightmare distress significantly predicted depression, *β* =  2.58, t =  9.22, *p* <  0.001, *R*^*2*^ (adjust) =  0.49, while at the same time autobiographical memory specificity did not predict depression, *β* =  -0.977, t =  -1.57, *p* =  0.12. Thus, these results suggested that nightmare distress mediated the effect of autobiographical memory specificity on depression. The mediating model of the mediating effect was in Fig.1.

Besides, results showed that the low autobiographical memory specificity group had a similar frequency of bizarre dreams to the high autobiographical memory specificity group (χ^2^ = .14, p = .71). By contrast, the former group’s dreams had more impacts on one’s life story than the latter group (Z =  2.33, p = .02). In addition, Spearman correlations showed that the impact of dreams on one’s life story was correlated with nightmare distress (r =  0.50, p <  0.001). By contrast, the former variable was not correlated with dream bizarreness (r =  -.15, p = .11).

## 4. Discussion

In line with our expectation, our results suggested that nightmare distress mediated the effect of autobiographical memory specificity on depression. The effect of autobiographical memory specificity on depression and the effect of nightmare distress on depression were supported by previous research (see introduction section). Yet no study had explored the relationship between autobiographical memory specificity and nightmare distress before. Thus, although we found that autobiographical memory specificity was correlated with nightmare distress, there was still a lack of direct evidence that the former variable could affect the latter variable. Nevertheless, as stated in the introduction section, two variables may be used to predict the effect of autobiographical memory specificity on nightmare distress. One was dream bizarreness, and the other was the impact of dreams on one’s life story. Our results suppoted the effect of the latter variable on this topic (see below). Thus, in our view, the mediating effect above was reliable. As stated in the introduction section, previous evidence showed that autobiographical memory specificity can affect depression (see section 1.1), and there was a lack of explanation for this effect. Our results suggested that the effect of autobiographical memory specificity on depression was based on nightmare distress. This finding may provide some insights and ideas for the area of clinical research which focused on the effect of autobiographical memory specificity on depression. For example, for depressive people, compared with interventions targeting impairments in reduced autobiographical memory specificity of these individuals, paying more attention on the intervention concerning nightmare distress may bring out more direct improvement in depression.

In the following, we talked about results related to dream bizarreness and results related to the impact of dreams on one’s life story separately. In this study, 51.8% of the most recent dream was the bizarre dream, which was measured by the rationality of a dream, or the everydayness of a dream. By contrast, two studies adopted a similar method found a lower frequency of the target dream (range from 24% to 30.2%) [13,26], although the target dream in those studies was rated by randomly summing two of the following three variables: the rationality, the everydayness, and the emotionality. In our opinion, questions for nightmares may affect participants’ recalling for the most recent dream, and as nightmares may be more bizarre than non-nightmares [20], here our results showed a higher frequency of the bizarre dream than previous research. In addition, contrary to our hypothesis, we did not find individual differences in autobiographical memory specificity was related to the frequency of bizarre dreams. It is argued that dream bizarreness may be related to the offline spreading activation process of autobiographical memory network, as different associated autobiographical memories may be put together in one dream narrative [12]. Furthermore, it is argued that the process may be affected by emotion, which may act as a marker to choose useful memories for a dream narrative [29]. From this viewpoint, the offline spreading activation process may be affected by emotion, which in turn may be related to dream bizarreness [29]. Although reduced autobiographical memory specificity caused more unassociated memories during the spreading activation process of autobiographical memory network [14], these unassociated memories may not affect emotions, and thus these unassociated memories may not be used during the formation of dream narrative. As a result, individual differences in autobiographical memory specificity may not affect dream bizarreness. In addition, we found that dream bizarreness was not correlated with nightmare distress. Previous evidence found that nightmares were more emotional and bizarre than bad dreams [20], which may imply that nightmare distress was related to dream bizarreness (see section 1.3.2). Yet the result of that study was obtained by the within-group comparison method where each participant’s own different kinds of dreams were compared with each other. By contrast, here our result was obtained by the between-group comparison method where different kinds of groups’ dreams were compared with each other. As nightmare distress was related to the impact of dreams on one’s life story (see below), nightmare distress may be affected by individual differences in the sensitivity to negative stimuli, and thus the individual differences may affect our result for the relationship between dream bizarreness and nightmare distress.

Besides, we found that individual differences in autobiographical memory specificity was related to the impact of dreams on one’s life story. This result was in line with our anticipation where we assumpted that sometimes the effect of specific autobiographical memories on the life story may decrease the effect of dreams on the life story (section 1.3.1), and thus the result supported the assumption. In addition, the result also suggested that the impact of dreams on one’s life story was a trait-like variable. Currently, some research explored potential effects of dreams on waking life [30]. Our results here may suggest that individual differences in the impact of dreams on one’s life story was a factor that should be taken into consideration by this kind of research. In addition, we found that the impact of dreams on one’s life story was correlated with nightmare distress. This result was also in line with our anticipation (section 1.3.1). Concerning the topic of nightmare distress, one model focuses on the idea that nightmare distress is related to the dysfunction of emotional regulation during dreaming [6], while another model argues that nightmare distress is related to individual differences in the sensitivity to be affected by internal and external environment [31]. Here our result may support the latter kind of model, as individual differences in the impact of dreams on one’s life story may be a case that was related to the sensitivity to be affected by internal environment. Taken above results together, our results suggested that the relationship between autobiographical memory specificity and nightmare distress was related to the impact of dreams on one’s life story.

## 4.1. Conclusions

It has long been argued that autobiographical memory specificity can affect depression. Yet it was unclear the mechanism for this effect. Here our results showed that nightmare distress was the mediator for the effect. This finding may provide some insights and ideas for the area of clinical research which focused on the effect of autobiographical memory specificity on depression.

In addition, we found that individual differences in autobiographical memory specificity was related to the impact of dreams on one’s life story. This result suggested that research concerning the topic of the effect of dreams on waking life should consider individual differences in the impact of dreams on one’s life story, and the result also suggested that sometimes the effect of specific autobiographical memories on one’s life story may decrease the effect of dreams on one’s life story. In addition, we found that the impact of dreams on one’s life story was related to nightmare distress. This result may support the idea that nightmare distress is related to individual differences in the sensitivity to be affected by internal and external environment. Besides, these results suggested that the relationship between autobiographical memory specificity and nightmare distress was related to the impact of dreams on one’s life story.

## Appendix

### Rationality Scale

The considerations in scoring dream content under this category are the degree of likelihood of their occurrence, and the degree of their adherence to natural law.

4. Rational, and not unlikely. Examples: riding a bike; hitting a stone; falling off.

3. Rational possible (possible, conceivable, but uncommon or unexpected). Examples: being chased, caught, and raped; San Francisco being bombed by the Russians.

2. Rational unlikely (very unlikely, although not violating any natural law). Examples: being chased from tree to tree by a white bear; some men chased, caught, and tried to poison me.

1.5. Borderline (the operation of natural law is uncertain and questionable). Examples: a long row of black box-cars rolling by a railroad track; there being no engine.

1. Non-rational but comprehensible. Examples: playing in a barnyard and suddenly covered with green snakes; our guns wiped out everything in front of them.

0. Irrational (impossible in reality). Examples: a toothed fish chased me out of the pool and across the fields; about a man with a lion’s head.

B. Bizarre: for example, the veins on my chest stood out, studded with rhinestones and sequins.

### Everydayness Scale

The consideration in scoring dream content under this category is the degree to which the dream content approximates that of everyday life.

4. For dreams just like everyday life. Examples: making plans with a friend for a car trip to an eighboring town; having to go to the bathroom; working or talking with some people.

3. Slight variations from everyday life. Examples: running in a relay race with two best friends, somehow got in wrong exchange area and have to give up the race; or (a student) “I had already graduated and gotten a good position in my field.”

2. Unlikely variationsfrom everyday life. Examples: returning to apartment to find all the furniture gone and workmen removing the bathroom pipes; all the girls in the dorm getting together for the last time before vacation, and all sad and crying at the prospect of the long separation.

1.5. With an impossible twist to everyday life. Examples: cleaning out a fishbowl, the fish swim up the stream of water pouring into it; a horse performing tricks suddenly turns into an elephant.

1. Very unlikely in everyday life. Examples: walking along a dirt road, an airline flies so low over us we could almost touch it. It circles back, lands on the road hitting a group of people as though intentionally.

0. Very remote from everyday life, or with the feeling tone of the strange and unfamiliar. Examples: (1) three priests with icepicks sitting at a round table, each begins lightly pricking the left arm of his neighbor, increasing this to jabbing and furiously stabbing till it’s a horrible bloody scene; (2) “I walk through a maze of high hedges. I am trying to reach the centre. There is a mist in the air, and grass is beneath by feet. I feel I am near a river or a moat. I have very long hair, and clothes that belong to another century. I sing the old folk song, ‘Where I come from nobody knows.’ I feel I must get out or get to the center.”

B. Bizarre. Example: The veins on my chest stood out, studded with rhinestones and sequins.

## Supporting information

S1 File Data-public. (SAV)
